# GABA administration improves liver function and insulin resistance in offspring of type 2 diabetic rats

**DOI:** 10.1038/s41598-021-02324-w

**Published:** 2021-11-30

**Authors:** Azadehalsadat Hosseini Dastgerdi, Mohammadreza Sharifi, Nepton Soltani

**Affiliations:** 1 Department of Physiology, School of Medicine, Isfahan University of Medical Sciences, Isfahan, Iran; 2 Department of Genetics and Molecular Biology, School of Medicine, Isfahan University of Medical Sciences, Isfahan, Iran

**Keywords:** Endocrine system and metabolic diseases, Endocrinology

## Abstract

This study investigated the role of GABA in attenuating liver insulin resistance (IR) in type 2 diabetes parents and reducing its risk in their descendants’ liver. Both sexes’ rats were divided into four groups of non-diabetic control, diabetic control (DC), GABA-treated (GABA), and insulin-treated (Ins). The study duration lasted for six months and the young animals followed for four months. Consequently, hyperinsulinemic-euglycemic clamp was performed for all animals. Apart from insulin tolerance test (ITT), serum and liver lipid profile were measured in all groups. Glycogen levels, expression of *Foxo1*, *Irs2*, A*kt2*, and *Pepck* genes in the liver were assessed for all groups. Overall, GABA improved ITT, increased liver glycogen levels and decreased lipid profile, blood glucose level, and HbA1c in parents and their offspring in compared to the DC group. GIR also increased in both parents and their offspring by GABA. Moreover, the expression of *Foxo1*, *Irs2*, *Akt2*, and *Pepck* genes improved in GABA-treated parents and their descendants in compared to DC group. Results indicated that GABA reduced liver IR in both parents and their offspring via affecting their liver insulin signaling and gluconeogenesis pathways.

## Introduction

Type 2 diabetes (T2D) is characterized by insulin resistance (IR). Following the decline of insulin production and pancreatic beta (β)-cell failure, there is a decrease in glucose transport into the muscle and fat cells, as well as the liver. An increase in fat breakdown happens due to hyperglycemia^1^.

The liver is a vital organ in glucose metabolism^2^. This organ creates a balance between the glucose uptake and storage of glycogenesis, gluconeogenesis, and glycogenolysis^3^. The secretion of insulin from the endocrine pancreas into the portal vein makes the liver 2–3 times more exposed to insulin concentrations. Also, insulin rapidly and potently reduces hepatic glucose production (HGP)^4^. Moreover, extreme production of hepatic glucose would contribute to fasting hyperglycemia in diabetes^5,6^. In the target cells of insulin action, many genes are involved in the insulin-signaling pathways. Among many of the molecules involved in the intracellular insulin signal processing in the liver, the genes protein kinase B (PKB, also known as *Akt2*)^7^, insulin receptor substrate 2 (*Irs2*)^8^, forkhead transcription factor 1 (*Foxo1*)^6,9^, and phosphoenolpyruvate carboxykinase (Pepck)^9^ play important roles in the regulation of glucose metabolisms of the liver. The insulin signaling pathway is generally thought to proceed Irs1 and Irs2. This leads to activation of phosphoinositide 3-kinase (PI3K), which phosphorylates and activates Akt2, and Akt2 induces insulin effects by activating other downstream pathways^10^. Pepck is an essential enzyme of gluconeogenesis by catalyzing the conversion of oxaloacetate (OAA) to phosphoenolpyruvate (PEP)^11^. Increases transcription of important glucoseogenesis enzymes, including Pepck, increases hepatic glucose production. Foxo1 leads to dephosphorylation of Akt2 and induces transcription of Pepck gluconeogenic enzyme and increases hepatic glucose production^6^.

Moreover, IR is the best predicting index for the development of diabetes in offspring of T2D subjects. Previous studies have indicated an increase in the concentration of free fatty acids (FFAs) among descendants of parents with IR^12,13^. Therefore, the idea of IR being related to the dysregulation of intramyocellular fatty acid metabolism, in descendants of T2D parents, is most likely due to a genetic defect in mitochondrial oxidative phosphorylation ^13^.

GABA is made from glutamate by the enzyme glutamic acid decarboxylase and is the main inhibitory neurotransmitter in the central nervous system (CNS)^14^. GABA co-exists with insulin in the pancreatic β-cells and acts as an anti-diabetic factor^15^. In T2D patients, a significant reduction was observed in the circulating GABA concentrations^16^. GABA has an anti-diabetic effect by acting on beta cell proliferation and the immune system^17^. Previously, it was shown that GABA improved hyperglycemia in STZ-induced diabetic rats and in the male model of high-fat diabetes by reducing the gluconeogenesis^18,19^. However, this effect was not observed in their offspring^18^. It has been reported that GABA was involved in diabetes progression by regulating the insulin and glucagon processes as well as the glucose homeostasis^15^.

Many pieces of evidence have indicated the regulation of hepatic metabolic pathways in a mammalian liver by GABA^20^. Therefore, this study aimed to investigate whether GABA administration during pregnancy and lactation could reduce IR and decrease the defective expression of *Irs2*, *Akt2*, *Foxo1*and *Pepck* genes in the offspring of T2D rats.

## Material and methods

### Experimental animals

Twenty-eight male and female Wistar rats (initial weight: 120–150 g) were obtained. The rats were housed under controlled humidity (50% ± 5%) and light conditions (12 h light/dark cycle; lights on 07:00–19:00). All experiments and methods were performed in accordance with relevant guidelines and regulations. All animal experiments were performed in accordance with ARRIVE (Animal Research: Reporting of In Vivo Experiments) guidelines and approved by Isfahan University of Medical Science in Iran (No. IR.MUI.MED.REC.1398.582). All methods are reported in accordance with ARRIVE guidelines.

. Food and water were made available ad libitum and the room temperature was set at 23 ± 2° C. Cage size and colony grouping were completely similar across all groups. The animals were randomly divided into the following four groups (n = 7 each): the non-diabetic control (NDC) group in which the rats received a normal diet during the experiment; the diabetic control (DC) group, in which the rats received a high-fat diet (HFD) during the experiment; the Insulin-treated (Ins) group, in which the diabetic rats received a HFD and received 2.5 U/kg of insulin (Exir, Iran) twice per day intraperitoneally (IP); and eventually, the GABA-treated (GABA) group, in which the diabetic rats received a HFD and a 1.5 g/kg dose of GABA daily IP injection (Sigma-Aldrich, Hamburg, Germany)^19^.

### Experimental procedures

#### Diabetes induction

Groups were kept on a HFD for three months according to Table 1^10^. Subsequently, a 35 mg/kg dose of streptozotocin (STZ) was intraperitoneally injected to induce T2D. To confirm the T2D induction, an intraperitoneal insulin tolerance test (ITT) was performed and then blood sugar (BS) levels were evaluated^10^. Those rats with blood glucose levels above 250 mg/dl were considered as T2D cases^21^. The blood sugar level was measured via tail vein and by using a glucometer (Ascensia Elite XL, Germany). Also, the rats were weighed with a digital scale (WMF digital scale, France) in a weekly cycle. An ITT and food intake were performed every month as previously describe^19^.

#### Mating, delivery, and their descendants

The animals were permitted to mate in such a way that each male could couple with only one female to prevent genetic admixture. Subsequently, the male animals were separated and kept in their respective groups. Also, the female animals were retained under the same conditions as their respective groups during pregnancy and lactation phases. After the delivery and breastfeeding, both parents underwent clamp tests and blood samples were collected; moreover, their livers were isolated and stored at – 80 °C to measure the expression of genes. The number of male and female offspring born in different groups was different. However, among the offspring born in each group, 5 males and 5 females, except in the GABA group that there were 3 offspring in both sexes entered the experimental process. The male and female offspring were separated and grouped similarly to their parents. However, they did not receive medications and were kept on a normal diet for four months. Finally, after the clamp test, their blood samples were collected and their livers were isolated to measure the expression of genes**.**

#### Euglycemic-hyperinsulinemic clamp

At the end of the protocol, the rats were anesthetized with ketamine (100 mg/kg, IP) and xylazine (8 mg/kg, IP). The rat’s right jugular vein was cannulated with polyethylene tubing (PE 9658, Microtube Extrusions, North Rocks NSW, Australia) to infuse insulin and glucose. Moreover, the left carotid artery was exposed and cannulated for blood sampling^19^. Both cannulas were fixated to the rat’s neck to eliminate any possibility of being separated by the animal. The cannulas were heparinized and closed by a blocker.

After a 24-h recovery time, each animal was anesthetized with the inhalation of isoflurane. The jugular vein and carotid artery cannula were connected to the microinfusion pump (New Era Pump System Inc., Farmingdale, NY, USA). When the rat regained consciousness, a bolus of regular insulin (100 mU/kg) was first injected and then 20 mU/kg/min of it was immediately infused to keep the blood glucose at 100 ± 5 mg/dl for 30 min in the conscious rat. Hence, the glucose concentration was measured every 5–10 min and variable rates of glucose solution (25%) were infused to clamp glucose at euglycemia. Finally, the insulin-stimulated glucose uptake (during the last 30 min of the clamp) and the whole-body insulin sensitivity were evaluated by the glucose infusion rate (GIR)^22^.

#### Biomedical assay design

Serum alanine transaminase (ALT), aspartate transaminase (AST), serum and liver triglycerides (TG), low-density lipoprotein (LDL), as well as cholesterol concentrations were measured by rat ELISA kit (Mercodia AB: Uppsala, Sweden; Elisa kit: ZellBio GmbH, Germany) using an automatic plate reader (Stat fax-2100, Beiken Co.).

Calcium (Ca) and magnesium (Mg) levels in the serum and liver were assessed by the commercially available kits (Pish-Taz Teb, Tehran, Iran) and HbA1c was evaluated by the Greiner kits with the Arsenio method, using the manufacturer’s instructions and Hitachi 717 autoanalyzer (Roche Diagnostics, Basel, Switzerland).

Moreover, the ratio of glycogen to tissue weight was calculated as the glucose from enzymatic digestion; this was done by subtracting the free glucose (the homogenate incubated without enzyme) from the total amount of glucose in the liver homogenate. The levels of very-low-density lipoprotein (VLDL) in serum and liver were measured in all groups by the following formula: VLDL = TG/5^23^.

#### Expression of *Irs2*, *Akt2*, *Foxo1*, and *Pepck* genes

The total RNA was extracted using the Iraizol kit (RNA Anacell, Iran) and following the manufacturer’s instruction. A microgram (μg) of total RNA was used to synthesize complementary DNA (cDNA) via RB MMLV (RNA Anacell, Iran). Quantitative real-time polymerase chain reaction (RT-qPCR) was induced using SYBR-green in the real-time PCR machine (Applied Biosystem StepOne). One μL of total cDNA was mixed into 10 μL SYBR-Green-treated water and 10 pmol/mL of both forward and reverse primers for the measured genes. The primer sequences are represented in Table 2. The average expression of beta-actin was used as an internal reference gene and the comparative CT method was utilized to compute the relative expression value^24^.

**Table 1:** High fat diet composition.

Ingredients	Diet (g/kg)
Powdered NPD	365
Lard	310
Casein	250
Cholesterol	10
Vitamin and mineral mix	60
DL-Methionine	3
Yeast powder	1
Sodium chloride	1

**Table 2:** Primers sequences.

Primer	Forward	Reverse	Reference
*Irs2*	GCCACCGTGGTGAAAGAGTA	AGCGTTGGTTGGAAACATGC	Designed with NCBIs primer Blast
*Akt2*	CTGTTTCTGCGCGTGCTAC	CAGCATTAACACGCTGTCACC	Designed with NCBIs primer Blast
*Foxo1*	ACGAGTGGATGGTGAAGAGTG	CCTCCCTCTGGATTGAGCATC	Designed with NCBIs primer Blast
*Pepck*	CCCAAGAGCAGAGAGACACC	CATACATGGTGCGGCCTTTC	Designed with NCBIs primer Blast
*Beta actin*	ACAACCTTCTTGCAGCTCCTC	CTGACCCATACCCACCATCAC	Designed with NCBIs primer Blast

#### Statistical analysis

All data was presented as mean ± SEM. Comparison between groups was made by one-way and two-way ANOVA and Tukey’s post-hoc test. Moreover, a *p*-value less than 0.05 (*p* < 0.05) was considered statistically significant.

## Result

### Effect of GABA on blood glucose in parents and their offspring

**Figure 1:**
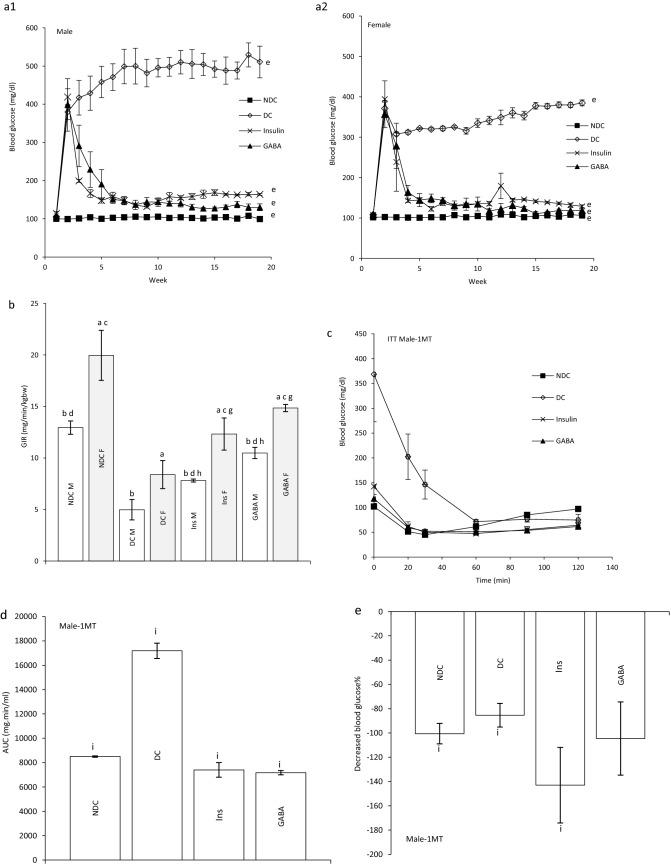

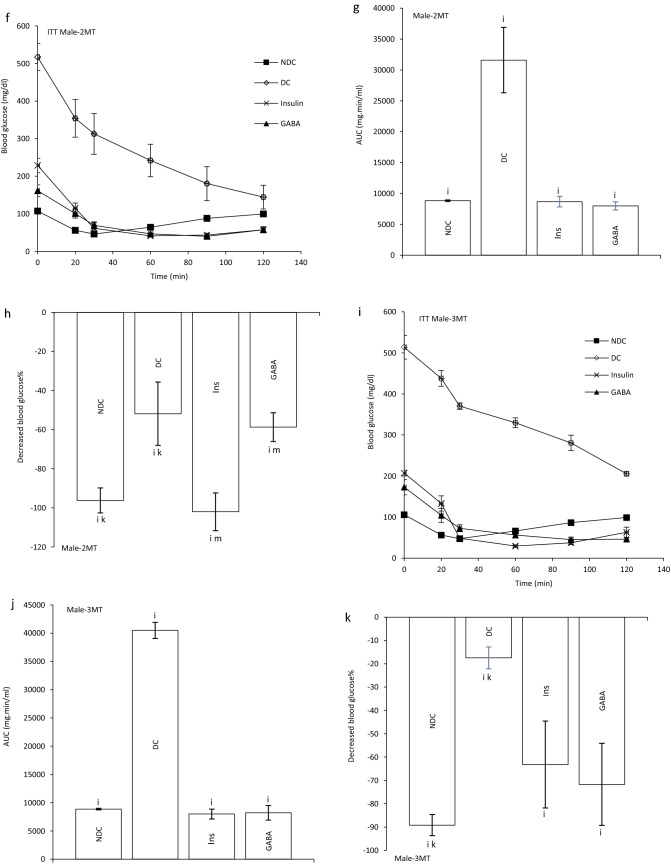

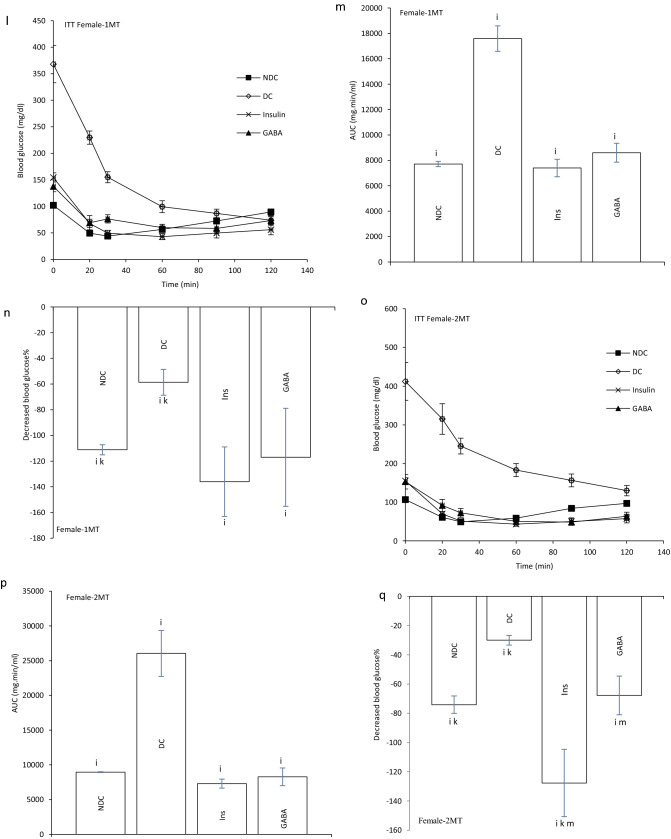

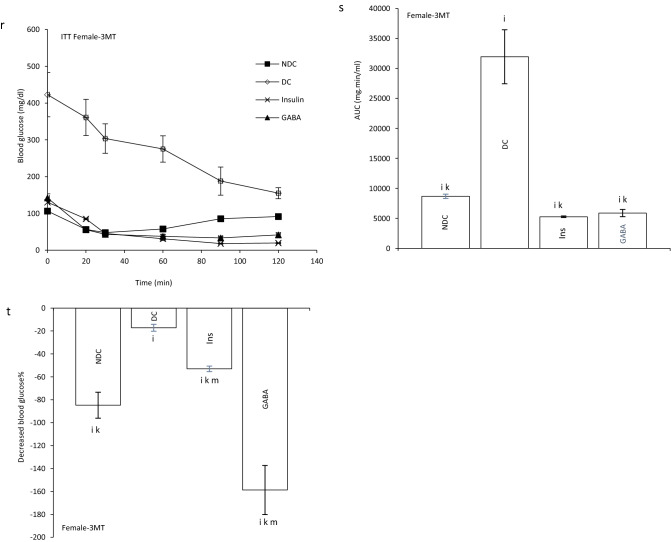
Comparison of blood glucose level in male (**a1**) and female (**a2**),GIR (**b**) and ITT in male 1 month (**c**), 2 months (**f**), 3 months (**i**) and their decreased blood glucose level (**e**, **h** and **k**) after intervention and the area under the glycaemic curve (AUC) for male (**d**, **g** and **j**) and also intraperitoneal insulin tolerance test (ITT) in female 1 month (**l**), 2 months (**o**), 3 months (**r**) and their decreased blood glucose level (**n**, **q** and **t**) after intervention and the area under the glycaemic curve (AUC) for female (m, p and s)in non-diabetic control group (NDC) was fed with normal diet, diabetic control group received high-fat diet and 35 mg/kg STZ (DC), diabetic animals treated with 1.5 gr/kg GABA via IP injection (GABA) and diabetic animals treated with insulin (2.5 U/kg twice per day) (Ins) . (7 rats in each group, data are expressed as mean ± S.E.M). (**e**) Significant difference in blood glucose level between DC group and other groups (*P* < 0.0001). (**a**) Significant difference in GIR between female DC group and other female groups (*P* < 0.001). (**b**) Significant difference in GIR between male DC group and other male groups (*P* < 0.01). (**c**) Significant difference in GIR between female NDC group and other female groups (*P* < 0.001). (**d**) Significant difference in GIR between male NDC group and other male groups (*P* < 0.001). (**g**) Significant difference in GIR between female GABA group and female Ins group (*P* < 0.01). (**h**) Significant difference in GIR between male GABA group and male Ins group (*P* < 0.01). (**i**) Significant difference in ITT between DC group and other groups (*P* < 0.0001). (**k**) Significant difference in ITT between NDC group and other groups (*P* < 0.001). (**m**) Significant difference in ITT between GABA group and Ins group (*P* < 0.001).

**Figure 2:**
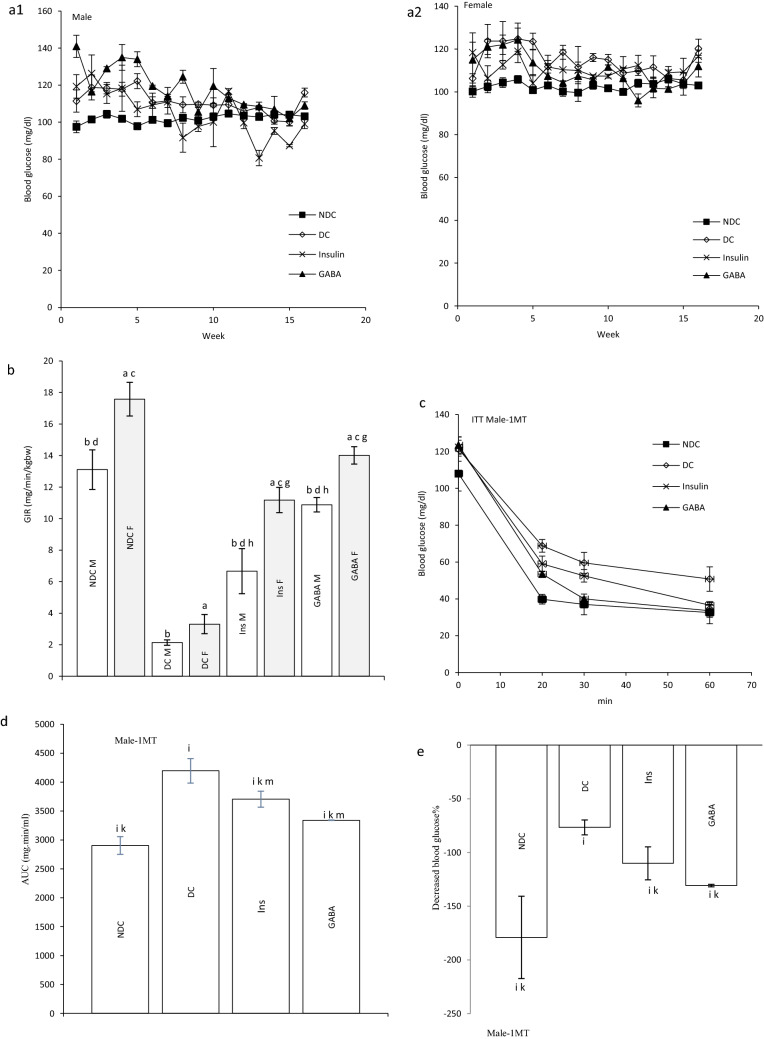

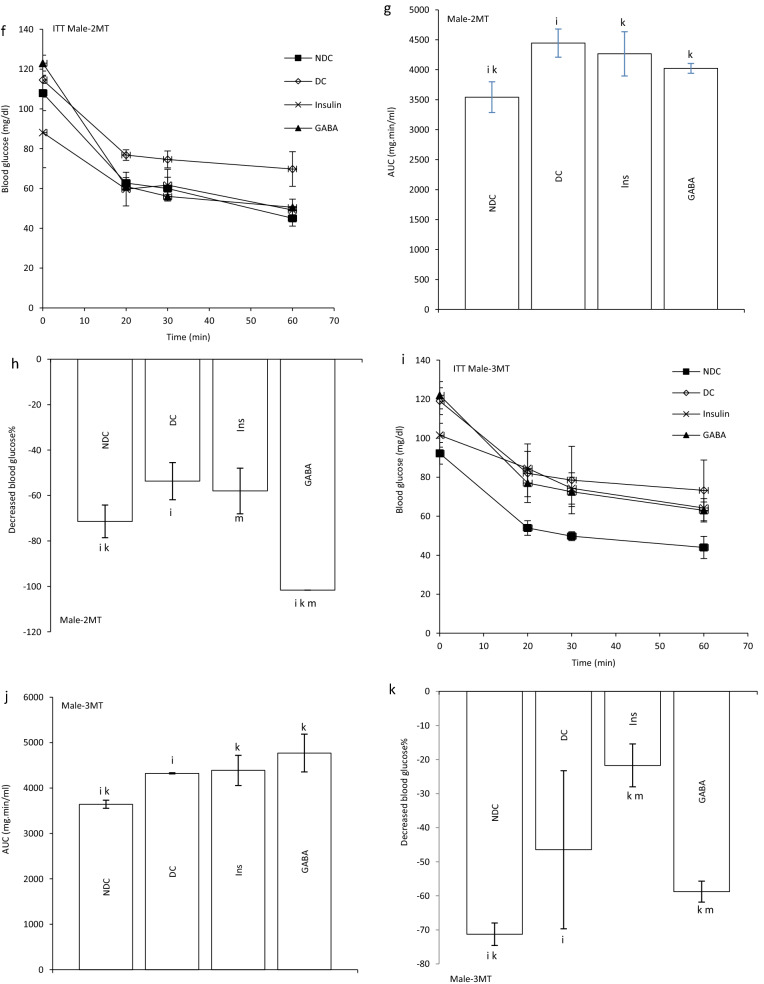

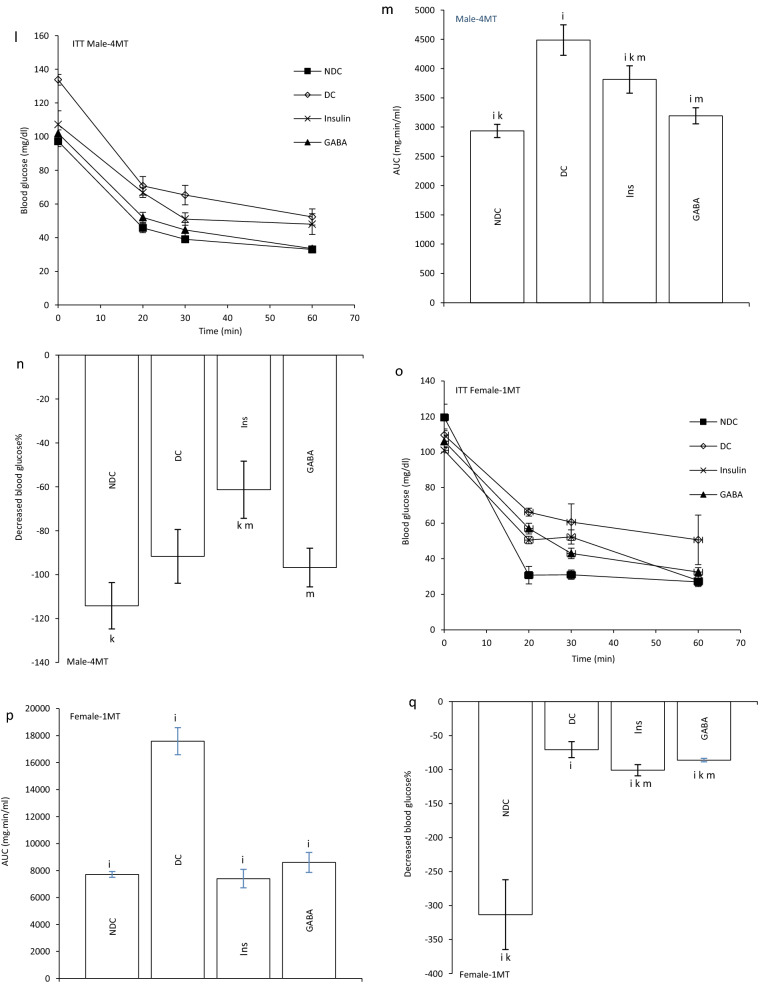

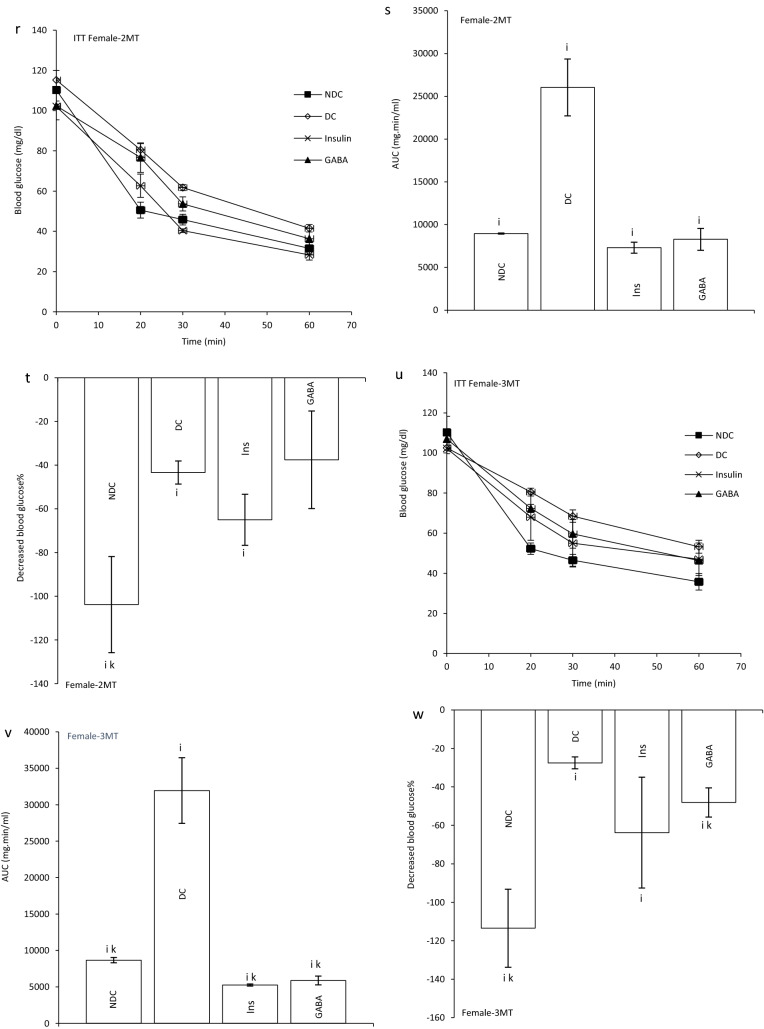

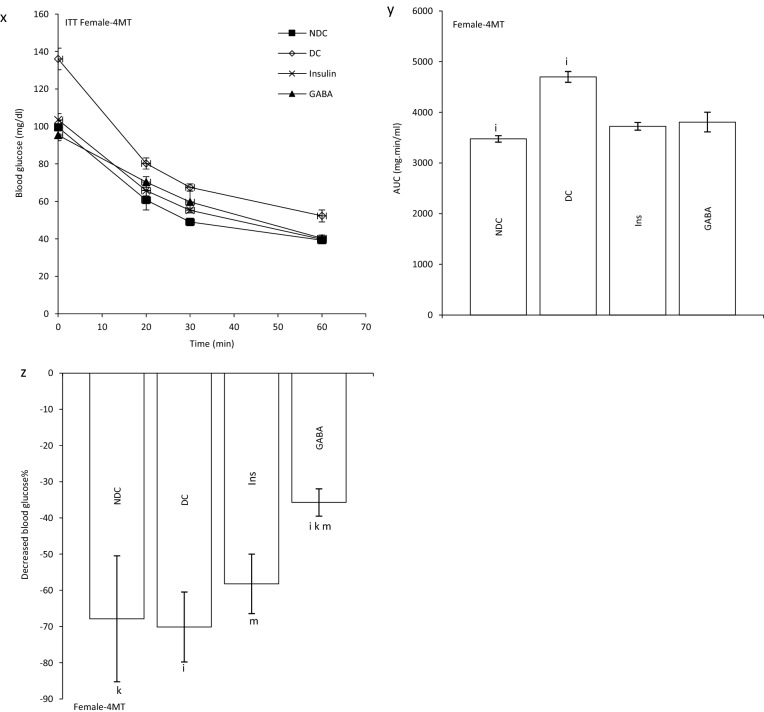
Comparison of blood glucose level in male (**a1**) and female offspring (**a2**),GIR in male and female offspring (**b**) and ITT in male offspring 1 month (**c**), 2 months (**f**), 3 months (**i**), 4 months(**l**) and their decreased blood glucose level (**e**, **h**, **k** and **n**) after intervention and the area under the glycaemic curve (AUC) for male offspring (**d**, **g**, **j** and **m**) and also intraperitoneal insulin tolerance test (ITT) in female offspring 1 month (**o**), 2 months (**r**), 3 months (**u**), 4 months (**x**) and their decreased blood glucose level (**q**, **t**, **w** and **z**)after intervention and the area under the glycaemic curve (AUC) for female offspring (**p**, **s**, **v** and **y**)in non-diabetic control group (NDC) was fed with normal diet, diabetic control group received high-fat diet and 35 mg/kg STZ (DC), diabetic animals treated with 1.5 gr/kg GABA via IP injection (GABA) and diabetic animals treated with insulin (2.5 U/kg twice per day) (Ins) . (3 rats in GABA group and 5 rats in other groups, data are expressed as mean ± S.E.M). (**a**) Significant difference in GIR between female DC group and other female groups (*P* < 0.001). (**b**) Significant difference in GIR between male DC group and other male groups (*P* < 0.01). (**c**) Significant difference in GIR between female NDC group and other female groups (*P* < 0.001). (**d**) Significant difference in GIR between male NDC group and other male groups (*P* < 0.001). (**g**) Significant difference in GIR between female GABA group and female Ins group (*P* < 0.01). (**h**) Significant difference in GIR between male GABA group and male Ins group (*P* < 0.01). (**i**) Significant difference in ITT between DC group and other groups (*P* < 0.0001). (**k**) Significant difference in ITT between NDC group and other groups (*P* < 0.001). (**m**) Significant difference in ITT between GABA group and Ins group (*P* < 0.001).

Diabetes induction led to increased plasma glucose concentration and blood glucose remained elevated 19 weeks after diabetes induction in males and females. Injection of GABA or insulin for 12 weeks significantly decreased plasma glucose concentrations in both sexes compared with DC groups, so that this reduction reached the level of the control group (male groups: NDC: 100 ± 0.44 mg/dl, DC: 498.75 ± 33.96 mg/dl, Ins: 162 ± 1.63 mg/dl, GABA: 134 ± 5.85 mg/dl; female groups: NDC: 104.6 ± 3.04 mg/dl, DC: 364.4 ± 23.7 mg/dl, Ins: 136.2 ± 8.31 mg/dl, GABA: 119 ± 3.02 mg/dl, *P* < 0.0001, Fig. 1a1, a2). The average of blood glucose levels was not significantly changed between the males and females offspring in all groups (Fig. 2a1, a2).

### Effect of GABA on the parents’ GIR and ITT

At the end of experiment hyperinsulinemic euglycemic clamp test was performed in all animals. Blood glucose was clamped at 100 ± 5 mg/dl. The GIR significantly decreased in DC groups in compare to NDC groups in both sexes (male groups: NDC: 12.95 ± 0.64 mg kg^−1^ min^−1^, DC: 4.97 ± 0.98 mg kg^−1^ min^−1^, *P* < 0.01; female groups: NDC: 19.95 ± 2.42 mg kg^−1^ min^−1^, DC: 8.38 ± 1.36 mg kg^−1^ min^−1^, *P* < 0.001). GABA and insulin therapy significantly increased the rate of GIR necessary to maintain euglycemia during the insulin infusion compared to the diabetic rats (male groups: DC: 4.97 ± 0.98 mg kg^−1^ min^−1^, Ins: 7.81 ± 0.14 mg kg^−1^ min^−1^, GABA: 10.48 ± 0.54 mg kg^−1^ min^−1^, *P* < 0.01; female groups: DC: 8.38 ± 1.36 mg kg^−1^ min^−1^, Ins: 12.32 ± 1.56 mg kg^−1^ min^−1^, GABA: 14.85 ± 0.35 mg kg^−1^ min^−1^, *P* < 0.001). Also, GABA and Ins groups showed a significant decrease in GIR compared to the NDC groups in both sexes (*P* < 0.001). This indicates that GABA and insulin, although able to reduce IR, have not been able to bring it to the control group. A significant increase was observed in GABA groups than Ins group in GIR, so it seems that GABA could decrease IR more than insulin in both sexes (*P* < 0.01) (Fig. 1b).

Our results showed that area under the curve (AUC) in ITT significantly decreased in GABA or Ins groups compared with DC group in both genders as much as the control group (male groups: first month: *P* < 0.0001; second months: *P* < 0.0001; third months: *P* < 0.0001); (female groups: first month: *P* < 0.0001, second months: *P* < :0.0001; third months: *P* < 0.0001). After 3 months also GABA and Ins female groups had lower AUC than NDC female groups (*P* < 0.001, Fig. 1c–t).

### Effect of GABA on the offspring’s GIR and ITT

GIR in male and female offspring of DC group significantly decreased in compare to the offspring of NDC groups (male groups: NDC: 13.10 ± 1.25 mg kg^−1^ min^−1^, DC: 2.13 ± 0.17 mg kg^−1^ min^−1^, *P* < 0.01; female groups: NDC: 17.57 ± 1.06 mg kg^−1^ min^−1^, DC: 3.30 ± 0.6 mg kg^−1^ min^−1^, *P* < 0.001). GIR in male and female offspring of GABA and Ins groups significantly increased in compare to the offspring of DC groups (male groups: DC: 2.13 ± 0.17 mg kg^−1^ min^−1^, Ins: 6.66 ± 1.4 mg kg^−1^ min^−1^, GABA: 10.87 ± 0.45 mg kg^−1^ min^−1^, *P* < 0.01; female groups: DC: 3.30 ± 0.6 mg kg^−1^ min^−1^, Ins: 11.17 ± 0.8 mg kg^−1^ min^−1^, GABA: 14.01 ± 0.54 mg kg^−1^ min^−1^, *P* < 0.001) while this increase was significantly (*P* < 0.01) grater in GABA offspring group than Ins offspring group in both sexes. Also, in male and female offspring of GABA and Ins groups a significant decrease in GIR was observed compared with male and female offspring of NDC groups (*P* < 0.001, Fig. 2b).

In male offspring of DC group, AUC in one, two, three and four months of born were significantly increased compared with male offspring of NDC group (*P* < 0.0001). While AUC significantly decreased in male offspring of Ins and GABA groups compared with male offspring of DC group only in first and fourth months of born (*P* < 0.0001). Decrease in AUC significantly was observed in male offspring of GABA group compared with male offspring of Ins group in first and fourth months of born (*P* < 0.001). In female offspring of DC group, AUC in the first to fourth months after birth were significantly increased compared with female offspring of NDC group (*P* < 0.0001). While AUC decreased in female offspring of Ins and GABA group in first, second and third month of born compared with female offspring of DC group (*P* < 0.0001). Also, in the third months after birth, a significant decrease in AUC was observed in the female offspring of Ins and GABA group compared with the female offspring of NDC group (*P* < 0.001, Fig. 2c–z).

### Changes of body weight in parents and their offspring

**Figure 3:**
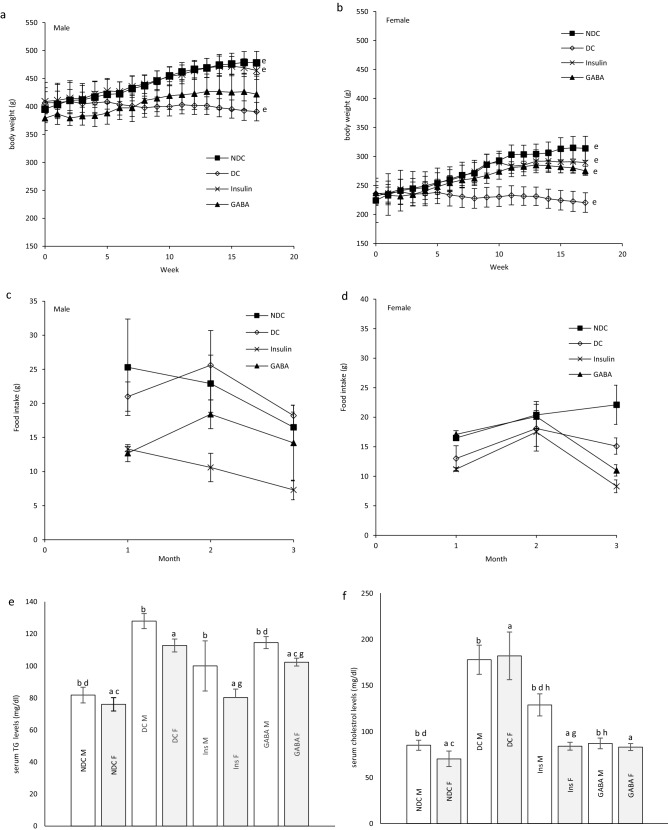

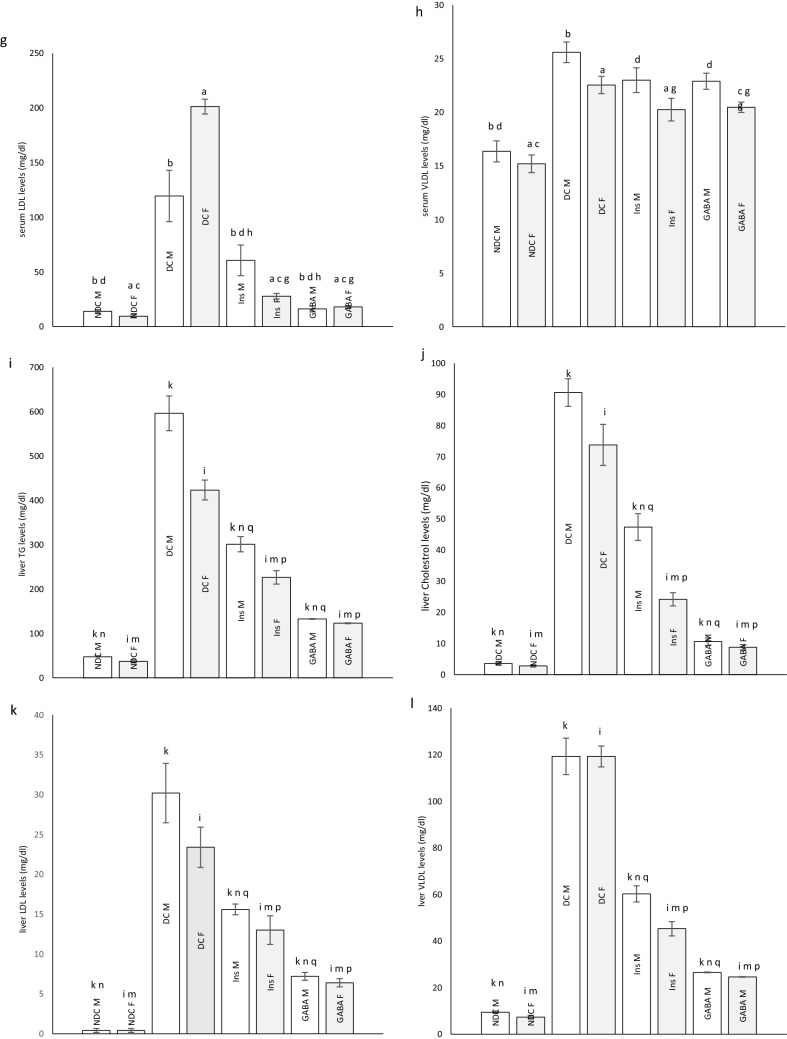
Comparison body weight in male (**a**) and female (**b**), food intake in male (**c**) and female (**d**), serum TG (**e**), cholesterol (**f**), LDL (**g**), VLDL (**h**) and liver TG (**i**), cholesterol (**j**), LDL (**k**), VLDL (**l**) in male and female 3 months after administration of GABA and insulin in non-diabetic control group (NDC) was fed with normal diet, diabetic control group received high-fat diet and 35 mg/kg STZ (DC), diabetic animals treated with 1.5 gr/kg GABA via IP injection (GABA), diabetic animals treated with insulin (2.5 U/kg/ml twice per day) (Ins).(7 rats in each group, data are expressed as mean ± S.E.M). (**e**) Significant difference in body weight between DC group and other female groups (*P* < 0.001). (**a**) Significant difference in serum lipid profile between female DC group and other female groups (*P* < 0.001). (**b**) Significant difference in serum lipid profile between male DC group and other male groups (TG: (*P* < 0.05); Cholesterol, LDL and VLDL: (*P* < 0.001)). (**c**) Significant difference in serum lipid profile between female NDC group and other female groups (*P* < 0.01). (**d**) Significant difference in serum lipid profile between male NDC group and other male groups (TG and VLDL: (*P* < 0.001); Cholesterol and LDL: (*P* < 0.01)). (**g**) Significant difference in serum lipid profile between female GABA group and female Ins group (*P* < 0.01). (**h**) Significant difference in serum lipid profile between male GABA group male Ins group (*P* < 0.01). (**i**) Significant difference in liver lipid profile between female DC group and other female groups (*P* < 0.001). (**k**) Significant difference in liver lipid profile between male DC group and other male groups (*P* < 0.001). (**m**) Significant difference in liver lipid profile between female NDC group and other female groups (TG, Cholesterol and LDL: (*P* < 0.01); VLDL: (*P* < 0.001)). (**n**) Significant difference in liver lipid profile between male NDC group and other male groups (TG, Cholesterol and LDL: (*P* < 0.01); VLDL: (*P* < 0.001)). (**p**) Significant difference in liver lipid profile between female GABA group and female Ins group (*P* < 0.001). (**q**) Significant difference in liver lipid profile between male GABA group male Ins group (*P* < 0.001).

**Figure 4:**
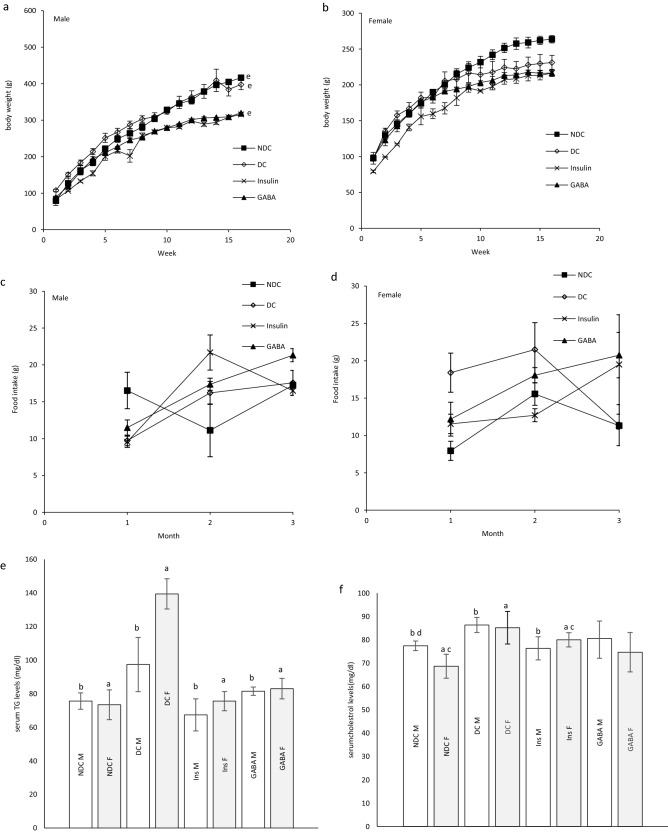

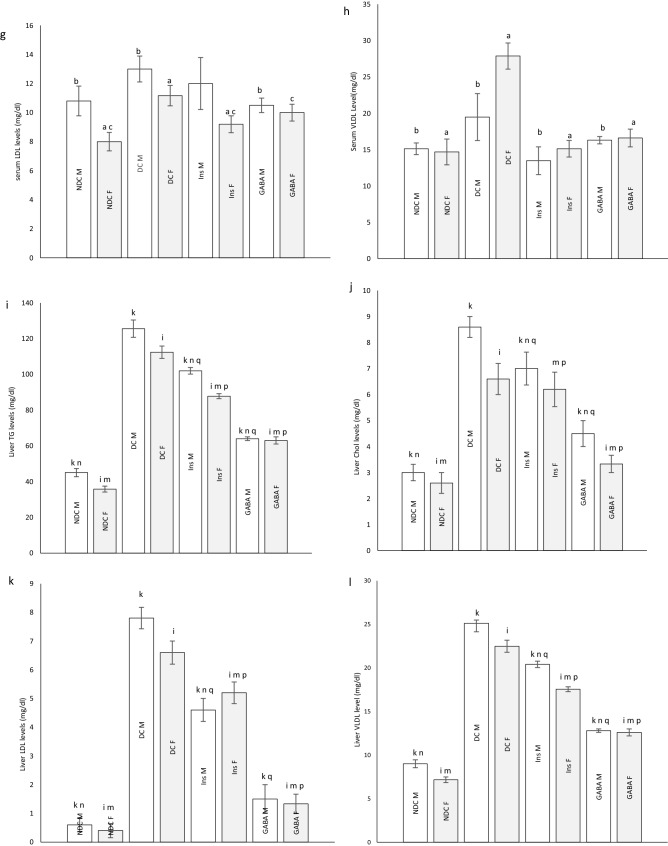
Comparison body weight in male (**a**) and female offspring (**b**), food intake in male (**c**) and female offspring (**d**), serum TG (**e**), cholesterol (**f**), LDL (**g**), VLDL (**h**) and liver TG (**i**), cholesterol (**j**), LDL (**k**), VLDL (**l**) in male and female offspring 3 months after administration of GABA and insulin in non-diabetic control group (NDC) was fed with normal diet, diabetic control group received high-fat diet and 35 mg/kg STZ (DC), diabetic animals treated with 1.5 gr/kg GABA via IP injection (GABA), diabetic animals treated with insulin (2.5 U/kg twice per day) (Ins).(3 rats in GABA group and 5 rats in other groups, data are expressed as mean ± S.E.M). (**e**) Significant difference in body weight between DC group and other female groups (P < 0.001). (**a**) significant difference in serum lipid profile between female DC group and other female groups (TG, Cholesterol and LDL: (*P* < 0.01); VLDL: (P < 0.001)). (**b**) Significant difference in serum lipid profile between male DC group and other male groups (TG and Cholesterol: (*P* < 0.01); LDL and VLDL: (*P* < 0.05)). (**c**) Significant difference in serum lipid profile between female NDC group and other female groups (Cholesterol: (*P* < 0.01); LDL: (*P* < 0.05)). (**i**) significant difference in liver lipid profile between female DC group and other female groups (TG, Cholesterol and VLDL: (*P* < 0.001); LDL: (*P* < 0.01)). (**k**) Significant difference in liver lipid profile between male DC group and other male groups (TG, LDL and VLDL: (*P* < 0.001); Cholesterol: (*P* < 0.01)). (**m**) Significant difference in liver lipid profile between female NDC group and other female groups (TG and Cholesterol: (*P* < 0.001); LDL: (*P* < 0.01)). (**n**) Significant difference in liver lipid profile between male NDC group and other male groups (TG and VLDL: (*P* < 0.001); Cholesterol and LDL: (*P* < 0.01)). (**p**) Significant difference in liver lipid profile between female GABA group and female Ins group (TG, LDL and VLDL: (*P* < 0.001); Cholesterol: (*P* < 0.01)). (**q**) Significant difference in liver lipid profile between male GABA group male Ins group (TG and VLDL: (*P* < 0.001); Cholesterol and LDL: (*P* < 0.01)).

Weight after 16 weeks in diabetic rats did not considerably change in neither sex, while in the NDC and Ins groups weight increased compared to the DC group in both sexes (*P* < 0.001), but in GABA treated animals the weight gain was significantly only observed in the female group (*P* < 0.001, Fig. 3a, b). Increasing body weight was observed in all groups of offspring during of the study. The body weight of male DC offspring showed a significant reduction in compared to the male NDC group (*P* < 0.001). While male offspring in the GABA group weighed less than male offspring in the DC group (*P* < 0.001, Fig. 4a). There was no significant difference between the groups of female offspring in body weight (Fig. 4b).

### The parent’s and their offspring food intake

There was no significant difference in food intake between the experimental parent groups (Fig. 3c, d) and their offspring (Fig. 4c, d).

### Parent’s HbA1c level

HbA1c in the DC groups significantly increased in compared to the NDC groups (male groups: NDC: 5.4% ± 0.05, DC: 5.8% ± 0.05; P < 0.01); female groups: NDC: 5.13% ± 0.17, DC: 5.63% ± 0.08; P < 0.001) but administration of GABA or insulin decreased HbA1c in diabetic rats in both sexes (male groups: DC: 5.8% ± 0.05, Ins: 5.23% ± 0.06, GABA: 5.06% ± 0.08, P < 0.01; female groups: DC: 5.63% ± 0.08, Ins: 5.2% ± 0.05, GABA: 4.93% ± 0.08, P < 0.001, Table 3).

**Table 3:** Comparison of serum biochemical factors in male and female groups of non-diabetic control group (NDC) was fed with normal diet, diabetic control group received high-fat diet for 3 months and 35 mg/kg STZ (DC), diabetic animals treated with 1.5 gr/kg GABA via IP injection (GABA) and diabetic animals treated with insulin (2.5 U/kg twice per day) (Ins) and their offspring.

Groups	Parents								Offspring							
Sexes	Male				Female				Male				Female			
Indexes	NDC	DC	Ins	GABA	NDC	DC	Ins	GABA	NDC	DC	Ins	GABA	NDC	DC	Ins	GABA
AST	116.2 ± 8.72^b,d^	246 ± 16.08^b^	159 ± 35.51^b,d^	181.5 ± 4.09^b,d^	213.5 ± 29.5^a,c^	394.25 ± 8.39^a^	212.5 ± 13.62^a,g^	161.6 ± 9.61^a,c,g^	252.6 ± 17.63^k^	280.33 ± 3.06^k^	196.83 ± 20.59^k^^,n^	261 ± 2^k^^,n^	153.66 ± 24.64^i^	230.83 ± 5.16^i^	148 ± 4.19^i,m^	218 ± 11.35^i,m^
ALT	82 ± 2.36^b,d^	137.5 ± 9.59^b^	83.4 ± 13.55^b^	85.5 ± 4.17^b^	59.75 ± 5.34^a,c^	140 ± 6.17^a^	67.5 ± 9.95^a^	81.6 ± 6.93^a,c^	63.2 ± 3.36^k^	75 ± 3.01^k^	69.83 ± 6.66	59 ± 3^k^	55.83 ± 7.95^i^	69.5 ± 3.64^i^	59 ± 4.61^i^	56.33 ± 2.4^i^
Mg	2.48 ± 0.12^b,d^	1.57 ± 0.19^b^	2.12 ± 0.1^b,d^	1.87 ± 0.08^b,d^	2.35 ± 0.12^a^	1.8 ± 0.09^a^	1.9 ± 0.21	2.06 ± 0.08^a^	2.52 ± 0.09^k^	2.3 ± 0.04^k^	2.35 ± 0.1	2.55 ± 0.05^k^	2.38 ± 0.13^i^	2.16 ± 0.06^i^	2.3 ± 0.04^i^	2.43 ± 0.14^i^
Ca	10.34 ± 0.12^b^	10.82 ± 0.11^b^	10.28 ± 0.5^b^	10.6 ± 0.19^b^	9.8 ± 0.15^a^	10.77 ± 0.17^a^	10.57 ± 0.2^ g^	9.83 ± 0.14^a,g^	10.18 ± 0.2^k^	10.53 ± 0.18^k^	9.41 ± 0.43^k^	9.95 ± 0.35^k^	10.08 ± 0.22	10.25 ± 0.05^i^	9.52 ± 0.29^i^	9.76 ± 0.06^i^
Ca/Mg	4.21 ± 0.21^b,d^	7.28 ± 1.11^b^	4.9 ± 0.36^b,d,h^	5.67 ± 0.18^b,d,h^	4.19 ± 0.17^a,c^	6.02 ± 0.25^a^	5.8 ± 0.71^c^	4.78 ± 0.28^a,c^	4.06 ± 0.14^k^	4.58 ± 0.05^k^	4.01 ± 0.13^k^	3.9 ± 0.06^k^	4.31 ± 0.29^i^	4.75 ± 0.15^i^	4.15 ± 0.18^i^	4.04 ± 0.23^i^
HbA1c	5.4% ± 0.05^b^	5.8% ± 0.05^b^	5.23% ± 0.06^b^	5.06% ± 0.08^b^	5.13% ± 0.17^a^	5.63% ± 0.08^a^	5.2% ± 0.05^a^	4.93% ± 0.08^a^	5.4% ± 0.05^k^^,n^	5.66% ± 0.08^k^	5.16% ± 0.17^k^^,n^	5.16% ± 0.03^k^^,n^	5.2% ± 0.05^i^	5.63% ± 0.06^i^	5.36% ± 0.03^i,p^	5.4% ± 0.5^i,p^

(3 rats in offspring of GABA groups and 5 rats in other groups, data are expressed as mean ± S.E.M).

*AST* aspartate aminotransferase, *ALT* alanine transaminase, *Mg* Magnesium, *Ca* calcium, *HbA1c* hemoglobin A1c.

^a^Significant difference in serum AST, ALT, Mg, Ca, Ca/Mg and HbA1c levels between female DC group and other female groups (AST, ALT, Ca and HbA1c: (*P* < 0.001); Mg: (*P* < 0.01); Ca/Mg: (*P* < 0.05)).

^b^Significant difference in serum AST, ALT, Mg, Ca, Ca/Mg and HbA1c levels between male DC group and other male groups (AST, Ca/Mg and HbA1c: (*P* < 0.01); ALT, Mg and Ca: (*P* < 0.001)).

^c^Significant difference in serum AST, ALT, Mg, Ca, Ca/Mg and HbA1c levels between female NDC group and other female groups (*P* < 0.05).

^d^Significant difference in serum AST, ALT, Mg, Ca, Ca/Mg and HbA1c levels between male NDC group and other male groups (Mg: (*P* < 0.001); Ca/Mg: (*P* < 0.05)).

^g^Significant difference in serum AST, ALT, Mg, Ca, Ca/Mg and HbA1c levels between female GABA group and female Ins group (*P* < 0.01).

^h^Significant difference in serum AST, ALT, Mg, Ca, Ca/Mg and HbA1c levels between male GABA group and male Ins group (*P* < 0.05).

^i^Significant difference in serum AST, ALT, Mg, Ca, Ca/Mg and HbA1c levels between female offspring DC group and other female offspring groups (AST, ALT, Mg, Ca and Ca/Mg: (*P* < 0.05); HbA1c: (*P* < 0.001)).

^k^Significant difference in serum AST, ALT, Mg, Ca, Ca/Mg and HbA1c levels between male offspring DC group and other male offspring groups (AST, ALT, Mg, Ca and Ca/Mg: (*P* < 0.05); HbA1c: (*P* < 0.01)).

m Significant difference in serum AST, ALT, Mg, Ca, Ca/Mg and HbA1c levels between female offspring NDC group and other female offspring groups (P < 0.05).

^n^Significant difference in serum AST, ALT, Mg, Ca, Ca/Mg and HbA1c levels between male offspring NDC group and other male offspring groups (*P* < 0.001).

^p^Significant difference in serum AST, ALT, Mg, Ca, Ca/Mg and HbA1c levels between female offspring GABA group and female offspring Ins group (*P* < 0.01).

### Offspring’s HbA1c level

HbA1c in male and female offspring of DC groups was higher than NDC groups (male groups: NDC: 5.4% ± 0.05, DC: 5.66% ± 0.08, *P* < 0.01; female groups: NDC: 5.2% ± 0.05, DC: 5.63% ± 0.06, *P* < 0.001), While HbA1c in male and female offspring of Ins and GABA groups was less than DC offspring group (male groups: DC: 5.66% ± 0.08, Ins: 5.16% ± 0.17, GABA: 5.16% ± 0.03, *P* < 0.01; female groups: DC: 5.63% ± 0.06, Ins: 5.36% ± 0.03, GABA: 5.4% ± 0.5, *P* < 0.001). In female offspring of GABA group significant increase in HbA1c was observed compared with female offspring of Ins group (*P* < 0.01). Male offspring of Ins and GABA groups showed a significant decrease in HbA1c compared to the male offspring of NDC group (*P* < 0.001), while in females this has not happened (Table 3).

### Serum TG, cholesterol, LDL and VLDL levels in parents

Diabetes significantly increased TG (male groups: (*P* < 0.05); female groups: (*P* < 0.001)), cholesterol, LDL and VLDL (*P* < 0.001) levels in parents in compared to NDC group. On the other hand insulin or GABA significantly improved the serum TG (male groups: (*P* < 0.05); female groups: (*P* < 0.001)), cholesterol and LDL (*P* < 0.001) of diabetic parents in compared to DC animals. VLDL decreased only in the Ins female group in compared to DC group (*P* < 0.001). Details of parental serum TG, cholesterol, LDL and VLDL changes in different groups are given in Fig. 3e–h.

### Serum TG, cholesterol, LDL and VLDL levels in offspring

Serum TG (*P* < 0.01), cholesterol (*P* < 0.01), LDL (male groups: (*P* < 0.05); female groups: (*P* < 0.01)) and VLDL (male groups: (*P* < 0.05); female groups: (*P* < 0.001)) levels in the male and female offspring of DC group were significantly increased in compared to the male and female offspring of NDC group. Offspring in GABA or Ins groups in both sexes showed a significant decrease in serum TG (*P* < 0.01) and VLDL (male groups: (*P* < 0.05); female groups: (*P* < 0.001)) levels in compared to offspring of DC groups. On the other hand, serum LDL levels in the female Ins group (*P* < 0.01) and male GABA group (*P* < 0.05) were significantly reduced in compared to the DC group. Details of offspring serum TG, cholesterol, LDL and VLDL changes in different groups are given in Fig. 4e–h.

### Liver TG, cholesterol, LDL and VLDL levels in parents

Diabetes caused an increase in liver TG, cholesterol, LDL and VLDL levels (*P* < 0.001) in DC parents in compared to the NDC parents. While administration of insulin or GABA in both sexes reduced liver TG, cholesterol, LDL and VLDL levels (*P* < 0.001) in compared to DC group. Treatment with GABA in both sexes significantly reduced liver TG, cholesterol, LDL and VLDL levels (*P* < 0.001) in compared to Ins group. Details of parental liver TG, cholesterol, LDL and VLDL changes in different groups are given in Fig. 3i–l.

### Liver TG, cholesterol, LDL and VLDL levels in offspring

Liver TG (*P* < 0.001), cholesterol (male groups: (*P* < 0.01); female groups: (*P* < 0.001)), LDL (male groups: (*P* < 0.001); female groups: (*P* < 0.01)) and VLDL (*P* < 0.001) levels in both sexes of offspring of diabetic parents were significantly higher than those of non-diabetic parents. Offspring of parents treated with insulin or GABA had lower levels of TG (*P* < 0.001), LDL (male groups: (*P* < 0.001); female groups: (*P* < 0.01)) and VLDL (*P* < 0.001) in their liver than offspring of DC parents. Insulin in males and GABA in both sexes significantly was able to lower cholesterol levels in offspring of diabetic parents (male groups: (*P* < 0.01); female groups: (*P* < 0.001)). Details of offspring liver TG, cholesterol, LDL and VLDL changes in different groups are given in Fig. 4i–l.

### Serum AST and ALT levels in parents

Serum AST (male groups: (*P* < 0.01); female groups: (*P* < 0.001)) and ALT (*P* < 0.001) levels in male and female DC groups were significantly increased in compared to the male and female NDC groups. Treatment with GABA or insulin in diabetic rats in both sexes decreased serum AST (male groups: (*P* < 0.01); female groups: (*P* < 0.001)) and ALT (*P* < 0.001) levels in compared to the DC group. Details of parental serum AST and ALT changes in different groups are given in Table 3.

### Serum AST and ALT levels in offspring

Serum AST and ALT levels in male and female offspring of DC parents were significantly increased in compared to the male and female offspring of NDC parents (*P* < 0.05). Overall offspring of DC parents that treated with GABA or insulin showed a significant decrease in serum AST levels in compared to the offspring of DC groups in both sexes (*P* < 0.05). Offspring of GABA group in both sexes and female offspring of Ins group showed a significant decrease in plasma ALT levels in compared to offspring of DC group (*P* < 0.05). Details of offspring serum AST and ALT changes in different groups are given in Table 3.

### Serum Mg, Ca and Ca/Mg levels in parents

The results of this study showed that the use of GABA in sexes and insulin in males improves serum Mg (male groups: (*P* < 0.001); female groups: (*P* < 0.01)), Ca (*P* < 0.01) and Ca/Mg (male groups: (*P* < 0.01); female groups: (*P* < 0.05)) levels in diabetic parents in compared to DC groups. Details of serum Mg, Ca and Ca/Mg changes in parents in all experimental groups are listed in Table 3.

### Serum Mg, Ca and Ca/Mg levels in offspring

Offspring of GABA in either sexes or female Ins groups showed a significant increase in plasma Mg levels in compared to the offspring of DC groups (*P* < 0.05). Male offspring of Ins parents and offspring of GABA group in both sexes showed a significant decrease in serum Ca level in compared to the male offspring of DC parents (*P* < 0.05). While administration of GABA or insulin by diabetic parents resulted a decrease in Ca/Mg in both sexes of their offspring in compared to offspring of DC group (*P* < 0.05). Details of serum Mg, Ca and Ca/Mg changes in offspring in all experimental groups are listed in Table 3.

### Mg, Ca, Ca/Mg and glycogen level in liver of parents

Liver Mg levels did not change significantly between parent’s experimental groups. Diabetes resulted in a significant increase in liver Ca (*P* < 0.05) and Ca/Mg (*P* < 0.01) levels in DC parents in compared to NDC parents. Insulin therapy in diabetic parents resulted a significant reduction in liver Ca levels in compared to the NDC parents. However, this reduction was seen only in males of GABA-treated diabetic parents (*P* < 0.05). Insulin or GABA therapy in both sexes reduced Ca/Mg level in the liver in compared to DC groups.

Diabetes significantly reduced glycogen stores in the liver of parents in compared to NDC groups (*P* < 0.01). Consumption of GABA or insulin in diabetic parents led to a significant increase in liver glycogen levels in diabetic parents in compared to DC group (*P* < 0.01). Details of liver Mg, Ca, Ca/Mg and glycogen changes in parents in all experimental groups are listed in Table 4.

**Table 4:** Comparison of liver biochemical factors in male and female groups of non-diabetic control group (NDC) was fed with normal diet, diabetic control group received high-fat diet for 3 months and 35 mg/kg STZ (DC), diabetic animals treated with 1.5 gr/kg GABA via IP injection (GABA) and diabetic animals treated with insulin (2.5 U/kg twice per day) (Ins) and their offspring.

Groups	Parents								Offspring							
Sexes	Male				Female				Male				Female			
Indexes	NDC	DC	Ins	GABA	NDC	DC	Ins	GABA	NDC	DC	Ins	GABA	NDC	DC	Ins	GABA
Mg	2.67 ± 0.04	2.52 ± 0.11	2.7 ± 0.09	2.62 ± 0.08	2.6 ± 0.08	2.6 ± 0.04	2.65 ± 0.06	2.57 ± 0.06	2.67 ± 0.07	2.72 ± 0.07	2.85 ± 0.08	2.8 ± 0.1	2.62 ± 0.04^m^	2.55 ± 0.15	2.82 ± 0.11^p^	2.26 ± 0.17^m^^,p^
Ca	0.92 ± 0.13^b,d^	1.47 ± 0.11^b^	1.27 ± 0.02^b,d,h^	0.92 ± 0.08^b,h^	0.85 ± 0.5^a^	1.17 ± 0.13^a^	0.92 ± 0.04^a^	0.87 ± 0.06	0.8 ± 0.07^k^^,n^	1.47 ± 0.22^k^	1.6 ± 0.38^n q^	0.9 ± 0.1^k^^,q^	0.97 ± 0.24^i^	1.45 ± 0.18^i^	1.32 ± 0.17^p^	0.76 ± 0.17^m^^,p^
Ca/Mg	0.34 ± 0.04^b,d^	0.59 ± 0.06^b^	0.47 ± 0.01^b,d,h^	0.35 ± 0.03^b,h^	0.32 ± 0.02^a^	0.45 ± 0.05^a^	0.34 ± 0.02^a^	0.33 ± 0.04^a^	0.29 ± 0.01^k^^,n^	0.53 ± 0.07^k^	0.55 ± 0.11^k^^,n,q^	0.32 ± 0.02^k^^,n,q^	0.28 ± 0.02^i,m^	0.56 ± 0.04^i^	0.46 ± 0.05^i,m,p^	0.33 ± 0.05^i,m,p^
Gly	0.46 ± 0.07^b,d^	0.03 ± 0.00^b^	0.08 ± 0.00^b,d,h^	0.15 ± 0.04^b,d,h^	0.31 ± 0.03^a,c^	0.02 ± 0.00^a^	0.05 ± 0.00^a,c,g^	0.1 ± 0.00^a,c,g^	0.43 ± 0.15^k^^,n^	0.04 ± 0.00^k^	0.04 ± 0.00^n,q^	0.1 ± ± 0.01^k^^,n,q^	0.43 ± 0.1^i,m^	0.05 ± 0.02^i^	0.02 ± 0.00^m^^,p^	0.09 ± 0.00^i,m,p^

(3 rats in offspring of GABA groups and 5 rats in other group, data are expressed as mean ± S.E.M).

*Mg* magnesium, *Ca* calcium, *Gly* Glycogen.

^a^Significant difference in liver Mg, Ca, Mg/Ca and Gly levels between female DC group and other female groups (Ca: (*P* < 0.05); Ca/Mg and Gly: (*P* < 0.01)).

^b^Significant difference in liver Mg, Ca, Mg/Ca and Gly levels between male DC group and other male groups (Ca: (*P* < 0.05); Ca/Mg and Gly: (*P* < 0.01)).

^c^Significant difference in liver Mg, Ca, Mg/Ca and Gly levels between female NDC group and other female groups (Ca: (*P* < 0.05); Gly: (*P* < 0.001)).

^d^Significant difference in liver Mg, Ca, Mg/Ca and Gly levels between male NDC group and other male groups (Ca: (*P* < 0.05); Ca/Mg: (*P* < 0.01); Gly: (*P* < 0.001)).

^g^Significant difference in liver Mg, Ca, Mg/Ca and Gly levels between female GABA group and female Ins group (*P* < 0.05).

^h^Significant difference in liver Mg, Ca, Mg/Ca and Gly levels between male GABA group and male Ins group (Ca: (*P* < 0.001); Ca/Mg: (*P* < 0.01); Gly: (*P* < 0.05)).

^i^Significant difference in liver Mg, Ca, Mg/Ca and Gly levels between female offspring DC group and other female offspring groups (Ca: (*P* < 0.05); Ca/Mg: (*P* < 0.001); Gly: (*P* < 0.01)).

^k^Significant difference in liver Mg, Ca, Mg/Ca and Gly levels between male offspring DC group and other male offspring groups (*P* < 0.01).

^m^Significant difference in liver Mg, Ca, Mg/Ca and Gly levels between female offspring NDC group and other female offspring groups (Mg and Gly: (*P* < 0.01); Ca/Mg: (*P* < 0.001)).

^n^Significant difference in liver Mg, Ca, Mg/Ca and Gly levels between male offspring NDC group and other male offspring groups (Ca: (*P* < 0.05); Ca/Mg and Gly: (*P* < 0.001)).

^p^Significant difference in liver Mg, Ca, Mg/Ca and Gly levels between female offspring GABA group and female offspring Ins group (Mg and Ca: (*P* < 0.01); Ca/Mg and Gly: (*P* < 0.001)).

^q^Significant difference in liver Mg, Ca, Mg/Ca and Gly levels between male offspring GABA group and male offspring Ins group (Ca: (*P* < 0.05); Ca/Mg and Gly: (*P* < 0.001)).

### Mg, Ca, Ca/Mg and glycogen level in liver of offspring

Liver Mg levels in the female GABA group were significantly lower than female NDC and Ins groups. GABA therapy in diabetic parents. GABA therapy by diabetic parents significantly reduced liver Ca levels of male and female offspring in compared to the offspring of DC parents (male groups: (*P* < 0.01); female groups (*P* < 0.05)). Ca/Mg ratio in liver increased significantly in offspring of DC groups in compared to NDC groups (male groups: (*P* < 0.01); female groups: (*P* < 0.001)). GABA administration in parents reduced Ca/Mg level in the liver of their offspring in both sexes (male groups: (*P* < 0.01); female groups: (*P* < 0.001).

Diabetes resulted a significant decrease in liver glycogen levels in male and female offspring in compared to NDC groups (*P* < 0.01),while treatment of diabetic parents with GABA significantly increased liver glycogen levels in their male and female offspring in compared to DC groups (*P* < 0.01) (Table 4). Details of liver Mg, Ca, Ca/Mg and glycogen changes in offspring in all experimental groups are listed in Table 4.

### *Foxo1*,* Irs2*, *Akt2* and *Pepck* mRNA expressions in parents

**Figure 5:**
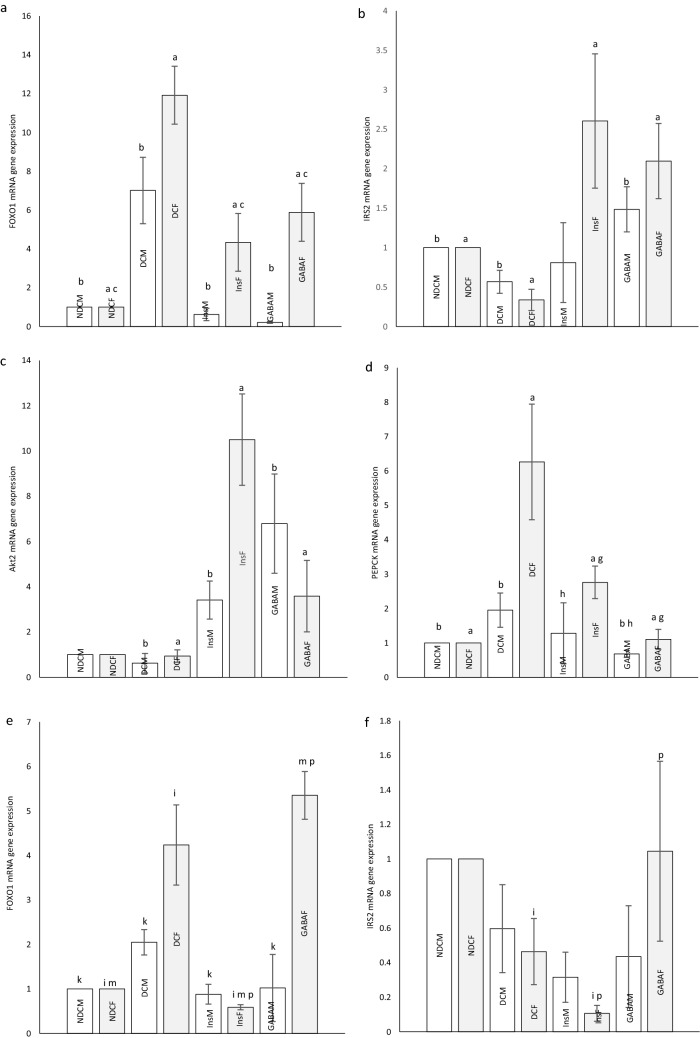

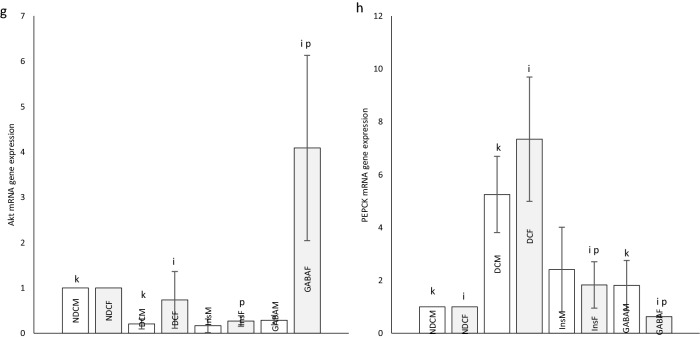
*Foxo1*, *Irs2*, *Akt2* and *Pepck* mRNA expression : Comparison *of Foxo1* (**a**), *Irs2* (**b**), *Akt2* (**c**) and *Pepck* (**d**) mRNA expression in male and female of non-diabetic control group (NDC) was fed with normal diet, diabetic control group received high-fat diet and 35 mg/kg STZ (DC), diabetic animals treated with 1.5 gr/kg GABA via IP injection (GABA), diabetic animals treated with insulin (2.5 U/kg twice per day) (Ins) and *Foxo1* (**e**), *Irs2* (**f**), *Akt2* (**g**)and *Pepck* (**h**) mRNA expression in their male and female offspring. (3 rats in offspring GABA groups and 5 rats in other groups, data are expressed as mean ± S.E.M). (**a**) Significant difference in *Foxo1*, *Irs2*, *Akt2* and *Pepck* mRNA expression between female DC group and other female groups (*Foxo1* and *Pepck*:(*P* < 0.001); *Irs2*: (*P* < 0.05); *Akt2*: (*P* < 0.01)). (**b**) Significant difference in *Foxo1, Irs2, Akt2 and Pepck* mRNA expression *b*etween male DC group and other male groups (*Foxo1*:(*P* < 0.001); *Irs2* and *Pepck*: (*P* < 0.05); *Akt2*: (*P* < 0.01)). (**c**) Significant difference in *Foxo1*, *Irs2*, *Akt2* and *Pepck* mRNA expression between female NDC group and other female groups (*P* < 0.001). (**g**) Significant difference in *Foxo1*, *Irs2*, *Akt2* and *Pepck* mRNA expression between female GABA group and female Ins group (*P* < 0.001). (**h**) Significant difference in *Foxo1*, *Irs2*, *Akt2* and *Pepck* mRNA expression between male GABA group male Ins group (*P* < 0.01). (**i**) Significant difference in *Foxo1*, *Irs2*, *Akt2* and *Pepck* mRNA expression between female offspring DC group and other female offspring groups (*Foxo1* and *Irs2:* (*P* < 0.01(; *Akt2* and *Pepck*: (*P* < 0.05)(. (**k**) Significant difference in *Foxo1, Irs2, Akt2 and Pepck* mRNA expression *b*etween male offspring DC group and other male offspring groups (*P* < 0.05(. (**m**) Significant difference in *Foxo1*, *Irs2*, *Akt2* and *Pepck* mRNA expression between female offspring NDC group and other female offspring groups (*P* < 0.05). (**p**) Significant difference in *Foxo1*, *Irs2*, *Akt2* and *Pepck* mRNA expression between female offspring GABA group and female offspring Ins group (*Foxo1* and *Irs2*:(*P* < 0.01); *Akt2* and *Pepck*: (*P* < 0.05)).

Diabetes led to a significant increase in *Foxo1* mRNA compared with NDC group in both sexes (*P* < 0.001). Treatment with insulin or GABA significantly resulted in *Foxo1* mRNA depletion in diabetic rats in both sexes (*P* < 0.001). *Foxo1* mRNA expression was higher in female Ins and female GABA groups than NDC group (*P* < 0.001), while in male groups it was not different from NDC group (Fig. 5a).

*Irs2* mRANA significantly decreased in DC group compared with NDC group in both sexes (*P* < 0.05). While in female Ins group and both sexes of GABA group, *Irs2* mRANA increased compared with DC groups (*P* < 0.05, Fig. 5b).

Insulin and GABA therapy significantly increased the *Akt2* mRNA in type 2 diabetic rats groups in both sexes compared with DC groups (*P* < 0.01, Fig. 5c).

*Pepck* mRNA significantly increased in DC group compared with NDC group in both sexes (male groups: *P* < 0.05; Female groups: *P* < 0.001), while *Pepck* mRNA in female Ins group and male and female GABA groups decreased compared with DC group (male groups *P* < 0.05; female groups: *P* < 0.001), The reduction of *Pepck* mRNA in the GABA group was significantly higher than in the Ins group in both sexes (Males: *P* < 0.01; Females: *P* < 0.001, Fig. 5d).

### *Foxo1, Irs2, Akt2* and *Pepck* mRNA expression in offspring

In offspring of DC groups, a significant increase in *Foxo1* mRNA were observed compared with NDC group in both sexes (male groups: *P* < 0.05; female groups: *P* < 0.01). While in offspring of Ins group in both sexes and male offspring of GABA group *Foxo1* mRNA significantly decreased compared whit offspring of DC group (male groups: *P* < 0.05; female groups: *P* < 0.01). In the GABA female group, a significant increase and in the Ins female group a significant decrease in *Foxo1* mRNA was observed compared to the female NDC group (*P* < 0.05). In the GABA female group, a significant increase in *Foxo1* mRNA was observed compared to the female Ins group (*P* < 0.01, Fig. 5e).

Female offspring of Ins group showed a decrease in the *Irs2* mRNA compared to the female offspring of DC group (*P* < 0.01). In other ways, the *Irs2* mRNA was significantly increased in the female offspring of GABA group compared to female offspring of Ins group (*P* < 0.01, Fig. 5f).

*Akt2* mRNA decreased in the male offspring of DC group compared with male offspring of NDC group (*P* < 0.05). *Akt2* mRNA in female offspring of GABA group significantly increased in compared with female offspring of DC and Ins groups (*P* < 0.05, Fig. 5g).

T2D significantly increased the *Pepck* mRNA in the male and female offspring (*P* < 0.05). Treatment of diabetic rats with insulin in females or GABA in both sexes reduced the *Pepck* mRNA in their female offspring (*P* < 0.05), this decrease was significantly greater in the female offspring of GABA group than female offspring of Ins group (*P* < 0.05, Fig. 5h).

## Discussion

The results of the present study indicated that treating diabetic parents with GABA or insulin improved IR and liver function in compared to non-treated diabetic animals. Our results showed that GABA beside improves blood glucose level and GIR, the golden standard method to measure IR, could improve serum and liver lipid profile, Mg and Ca levels, Ca/Mg ratio, serum ALT and AST levels, liver glycogen level, and expression of *Foxo1, Irs2, Akt2*, and *Pepck* genes in the liver among either one or both genders and their offspring. Our previous study showed that GABA could increase GIR in diabetic parents and their offspring, the muscles were not completely involved in this action^18^. So, this study aimed to investigate the possibility role of GABA or insulin in reducing the IR in the liver of T2D cases and their offspring.

Our results showed that T2D in both parents led to increase glucose level in the blood. Treatment with insulin or GABA could decrease blood glucose levels. These findings are agreement with previous findings^18,19^. However, in the mean of blood sugar among their descendants’ significant changes were not observed.

ITT and the hyperinsulinemic-euglycemic clamp test were used to evaluate IR throughout the body. Based on current study, after diabetes was induced the ITT results showed a significant increase in both parents at the first, second, and third months. Also, in their offspring (from diabetic parents) increase in ITT results was seen at the first to fourth months after birth. The use of GABA or insulin in diabetic rats in both genders reduced ITT at the first, second, and third months after the treatment. In previous studies, GABA had also improved the ITT among male diabetic rats^19^. Notably, the improvement of ITT was measured in the offspring of rats in the GABA group. The act of GABA is known as an endogenous insulin secretion enhancer; therefore, such act might contribute to the impairment of β-cell functions in diabetic patients^25^. Overall, the results of ITT in our study showed using of GABA in diabetic parents more effective in decreasing IR in both parents and their offspring.

In this study, GIR level was significantly decreased in diabetic rats and their offspring. Also, GABA or insulin therapy significantly increased GIR in diabetic parents and their offspring among both genders and the improvement rate caused by GABA therapy was greater than insulin treatment (particularly, in female rats). Ovarian hormones influence insulin sensitivity during the menstrual cycle, pregnancy, and menopausal transition. Moreover, estrogens could be considered as the regulator of body composition, energy balance, and insulin sensitivity in both genders^26^. In inevitably, estrogens may increase hepatic insulin sensitivity via reducing gluconeogenesis and glycogenolysis^27^ and not only prevent β-cell apoptosis from reducing the pro-inflammatory signaling, but also improve insulin action^28,29^. Other study has been shown that estrogens helped insulin production sustenance in both male and female diabetic mice^29^. The anti-diabetic role of ERα is also suggested by Ribas et al. (2010).

The results concerning GIR and ITT in this study showed that GABA or insulin administration in diabetic parents and their offspring were reduced their level of IR. The increasing GIR in this study is compatible with reduction of IR in previous study^18^. The partly effect of GABA or insulin in IR reduction was due to the enhancing insulin pathways in parents muscle tissues^18^, but this effect was not seen in their offspring.

According to the present results, T2D led to increase LDL, cholesterol, VLDL, and TG in serum and liver. Notably, these factors showed enhancements among offspring of DC parents (predominantly male rats). Studies have shown that STZ caused a significant increase in total cholesterol, TG, and LDL in serum^16^. The insulin deficiency probably changes lipid profile in serum due to metabolic complication of diabetes and IR is associated with impaired lipoprotein lipase activity^16^.

The increased levels of free fatty acid and glucose, regulating the liver VLDL outputs, elevated TG levels in the liver and inhibiting the apoprotein B (apoB) degradation may cause increased in assembly and secretion of VLDL^30^. Any change in VLDL metabolism could result in LDL synthesis^30^. Hepatic IR played a key role in the progression of nonalcoholic fatty liver disease (NAFLD) by altering the TG synthesis and transport, increasing the lipolysis rate in the adipose tissues, and the FFA transport to the liver^31^. Therefore, instead of synthesis stimulation, inhibiting the oxidation may have contributed to the accumulation of lipids in the liver^32^. The study of animal models indicated that metabolic complications, such as NAFLD, occurred to the offspring of dams with diet-induced obesity^33^. Also, the use of GABA or insulin reduced the serum TG level in diabetic male and female rats. In the current study, cholesterol level decreased only in the insulin group; moreover, the LDL level decreased in the female Ins and male GABA groups.

GABA has been reported as a positive regulator of antioxidant enzymes; it reduces ROS, cholesterol, and TG in humans. Moreover, it could boost the antioxidant status by reducing oxidative stress, lipid peroxidation, and ROS. Therefore, it plays a significant role in subsidizing the hyperlipidemia and hyperglycemic states^34^. The results of current experimental studies revealed that induction of experimental diabetes enhanced the lipid peroxidation processes in different tissues^16^. In this study, the levels of lipid profile in the liver were measured; administration of GABA and insulin reduced the levels of LDL, cholesterol, TG, and VLDL in the liver among diabetic parents and their offspring. However, GABA had a greater impact on reducing liver fat levels in compared to insulin. Moreover, the fat levels were lower in females than in male rats. In a different study, the GABA in brown rice extract, not only enhanced HDL, but also reduced TG levels and total cholesterol concentrations in the liver^35^. It is stated that GABA has protective effects on the liver and reduces lipid levels in liver and plasma^36^. In female cases, the liver metabolisms are regulated via estrogen receptors that decrease lipogenesis, gluconeogenesis, and fatty acid uptake. Simultaneously, they enhance lipolysis, secretion of cholesterol, and glucose catabolism. Notably, estrogens participate in maintaining the lipid and cholesterol balances and play protective roles against hepatic lipid accumulation via suppressing lipogenesis, lipid uptake, and cholesterol synthesis as well as by promoting lipolysis and cholesterol removal^37^.

GABA seems to play a key role in regulating food intake and body weight via controlling the excitability, plasticity, and neuronal activity synchronization in the frontal cortex^38^. Also, by acting on type A receptors in the brain nuclei, it could suppress appetite and prevent any weight gain^38^. According to a study, 17-beta-estradiol modulated GABA release and the effects of GABA on the brain have been shown^39^. Therefore, the rat’s weight gain might have been due to its modulatory role in the brain via GABA-treated estrogen in diabetic female rats.

The results indicated that diabetes led to increase liver enzymes (AST and ALT) in parents and their offspring. Elevated ALT levels, as an alternative sign of NAFLD, could help predict the emergence of diabetes and metabolic syndrome development^40^. Moreover, the increased activities of ALT and AST are associated with obesity and IR^41^. Our finding indicated that GABA or insulin administration reduced the levels of serum ALT and AST in diabetic rats and their offspring. The mean liver enzymes were lower in female rats in all groups. Accordingly, a study indicated higher levels of mean serum ALT and AST among males^42^. GABA has been shown to attenuate hyperglycemia-induced damages to the liver by improving serum AST and ALT activities, which may be attributed to the modulations in cellular polyamine levels. Nevertheless, based on current results liver injury improvement could be attributed to the role of GABA in minimizing the oxidative stress in the liver^16^. Increasing in serum ALT and AST levels is proposed as an alternative indicator of fatty liver and IR^43,44^. Moreover, elevated levels of liver enzymes in the offspring of diabetic parents also indicate their IR. The increase rate was lowered by insulin and GABA administration in diabetic parents.

The IR-associated Ca and Mg play opposing roles in cells^45^. In this study, the subjects with the lowest Ca levels had the least glucose concentrations and IR. Ca levels had a direct correlation with glucose concentrations and IR^46^. However, some studies have suggested that Mg deficiency was common in diabetic people and might be a preceding factor in IR and hyperinsulinemia^47,48^. Therefore, the increased plasma Ca level, decreased plasma Mg level, and consequently, increase the ratio of Ca to Mg, indicate to increase IR^45,49^. High cellular Ca/Mg ratio could affect metabolic syndrome disorders in various tissues, highlighting the potential significance of the Ca/Mg ratio in the etiology of IR in T2D subjects^50^. Overall, the results of the present study indicated an increase in the ratio of Ca/Mg in the serum and liver in the offspring of diabetic parents. This result agrees with other data concerning the IR. Using GABA in both parents and insulin in male parents led to a decline in the Ca/Mg ratio. Nonetheless, a decrease was observed in the serum Ca/Mg ratio in all (male and female) descendants of Ins and GABA groups, as well as in the liver Ca/Mg ratio in both diabetic parents and their offspring who were treated with insulin or GABA. It is shown that GABA_A_ receptor signaling, with excitatory actions, could trigger the mitochondrial release of Mg^51^. Therefore, administering GABA and insulin could reduce IR in diabetic parents and their descendants by reducing the serum and liver Ca/Mg ratio.

Other findings indicated that diabetes also reduced the liver glycogen stores in parents and their offspring. Glycogen synthesis operates as a key pathway for glucose disposal after insulin stimulation. As such, lack of liver glycogen increases the fat and liver IR; moreover, loss of its synthesis directs glucose towards producing fat. This phenomenon correlates with impaired hepatic insulin signaling and glucose disposal, leading to an increase in liver fat^52^. The decrease of glycogen content in the diabetic liver might be a direct consequence of those metabolic disorders in diabetes that are caused either by increased glycogenolysis or decreased ability to synthesize glycogen^53^. Also, the inverse relationship between liver TG and glycogen content was attributed to IR. Therefore, the reduction of liver glycogen in diabetic parents and their offspring corresponds with other results concerning hepatic IR in this study. Treatment with insulin or GABA increased the liver stores of glycogen in parents and their descendants; however, the increase rate by GABA was more than by insulin. Moreover, the glycogen levels in the liver were lower in female rats in comparison with the male ones. Hence, GABA administration in diabetic parents results in reducing liver glycogen; consequently, it leads to a decreased level of IR in the liver of diabetic parents and their descendants. Administration of estradiol and progesterone (particularly together) prevents hepatic gluconeogenesis and contributes to the accumulation of glycogen in the liver^54^. These differences might further raise the risk of hyperglycemia and hepatic IR in male subjects under conditions in which hepatic fat is increased and/or insulin production is insufficient^55^.

Also, diabetes increased the expression of *Foxo1* and *Pepck* genes and decreased *Akt2* gene expression. Diabetes reduced *Irs2* gene expression in parents and their offspring; this reduction was observed only in female offspring. A former study demonstrated that antagonizing or reducing hepatic *Foxo1* activity in mice with IR improved their glucose tolerance and insulin responsiveness greatly^55^. In contrast to the current results, the expression levels of *Pepck* and *G6Pase* (major gluconeogenic enzymes) were reduced in the obese dams^33^. A maternal HFD (during pregnancy) increased the risk of metabolic syndrome disorders for the offspring as they matured (regardless of their adulthood environmental factors). Thus, the fetus seems to have responded to the nutritional stress by programming its growth in a way that would lead to an increased risk of future metabolic disorders, such as IR, increased body fat mass, and reduced hypophagic effect of central insulin^32^. In a different study, female parents with a HFD demonstrated an elevated hepatic expression of gluconeogenic enzymes; moreover, the insulin signaling was disrupted in the liver of their offspring^56^. Results of the present study indicate that using of GABA or insulin in parents decreased expression of *the Foxo1* gene and led to increase the expression of *the Akt2* gene. Moreover, GABA increased the expression of *Irs2* gene and decreased the expression of *Pepck* gene. This effect of insulin was observed only in female subjects. In the next generation, GABA reduced the expression of the *Pepck* gene in both genders and suppressed the expression of the *Foxo1* gene in male subjects. On the other hand, using of GABA by parents increased the expression of the *Akt2* gene in their female offspring possibly due to dissimilar effects of GABA on each gender. Moreover, this leads to the occurrence of IR in their descendants. The uncertainty regarding the physiological roles of *Foxo1* in hepatic metabolism might be due to obtaining information from IR livers, in which unchecked *Foxo1* activation results in profound metabolic abnormalities in gluconeogenesis, lipid metabolism, and glycogen storage^55^. According to the result of a research study, the GABA_A_ receptor expression occurs in hepatocytes^57^. The effects of GABA on insulin signaling in the liver had not been studied before. According to current results, GABA plays a significant role in reducing the liver IR not only by increasing the expression of *Akt2* and *Irs2* genes, but also by decreasing the expression of *Pepck* and *Foxo1* genes; moreover, it inhibits hepatic gluconeogenesis. These results suggested that GABA intake, during pregnancy and lactation could affect the programming of genes in rat descendants. Consequently, it may enhance insulin sensitivity^58^.

## Conclusion

In this study, a high-fat diet along with the induction of type 2 diabetes (T2D) led to increased insulin resistance (IR) in parents and their offspring. The GABA administration greatly reduced the liver IR in parents by decreasing the expression of *Foxo1* and *Pepck* genes as well as increasing the expression of *Irs2* and *Akt2* genes. GABA reduced *Foxo1* genes in male offspring and improved *Akt2* in female offspring of diabetic parents. GABA, on the other hand, reduced *Pepck* gene expression in offspring of diabetic parents, thereby reducing IR. Conversely, GABA improved the lipid profile in the liver and serum of T2D parents and their offspring. GABA maybe help T2D patients to control their liver IR.

## Data Availability

The datasets used and/or analyzed during the current study are available from the corresponding author on reasonable request.
